# Molecular Signatures of CB-6644 Inhibition of the RUVBL1/2 Complex in Multiple Myeloma

**DOI:** 10.3390/ijms25169022

**Published:** 2024-08-20

**Authors:** Weijun Yi, Sebastian A. Dziadowicz, Rachel S. Mangano, Lei Wang, Joseph McBee, Steven M. Frisch, Lori A. Hazlehurst, Donald A. Adjeroh, Gangqing Hu

**Affiliations:** 1 Department of Microbiology, Immunology & Cell Biology, West Virginia University, Morgantown, WV 26505, USA; wy0003@mix.wvu.edu (W.Y.); sedziadowicz@mix.wvu.edu (S.A.D.); rachel.s.mangano@vanderbilt.edu (R.S.M.); lei.wang1@hsc.wvu.edu (L.W.); jcm00035@mix.wvu.edu (J.M.); 2 Lane Department of Computer Science & Electrical Engineering, West Virginia University, Morgantown, WV 26506, USA; 3 Division of Clinical Pharmacology, Departments of Medicine and Pharmacology, Vanderbilt University School of Medicine, Nashville, TN 37232, USA; 4 Department of Biochemistry and Molecular Medicine, West Virginia University, Morgantown, WV 26506, USA; sfrisch@hsc.wvu.edu; 5 Department of Pharmaceutical Sciences, School of Pharmacy, West Virginia University, Morganton, WV 26506, USA; lahazlehurst@hsc.wvu.edu; 6 WVU Cancer Institute, West Virginia University, Morgantown, WV 26506, USA

**Keywords:** multiple myeloma, molecular signatures, RUVBL1/2, CB-6644

## Abstract

Multiple myeloma is the second most hematological cancer. RUVBL1 and RUVBL2 form a subcomplex of many chromatin remodeling complexes implicated in cancer progression. As an inhibitor specific to the RUVBL1/2 complex, CB-6644 exhibits remarkable anti-tumor activity in xenograft models of Burkitt’s lymphoma and multiple myeloma (MM). In this work, we defined transcriptional signatures corresponding to CB-6644 treatment in MM cells and determined underlying epigenetic changes in terms of chromatin accessibility. CB-6644 upregulated biological processes related to interferon response and downregulated those linked to cell proliferation in MM cells. Transcriptional regulator inference identified E2Fs as regulators for downregulated genes and MED1 and MYC as regulators for upregulated genes. CB-6644-induced changes in chromatin accessibility occurred mostly in non-promoter regions. Footprinting analysis identified transcription factors implied in modulating chromatin accessibility in response to CB-6644 treatment, including ATF4/CEBP and IRF4. Lastly, integrative analysis of transcription responses to various chemical compounds of the molecular signature genes from public gene expression data identified CB-5083, a p97 inhibitor, as a synergistic candidate with CB-6644 in MM cells, but experimental validation refuted this hypothesis.

## 1. Introduction

RUVBL1 and RUVBL2, collectively referred to as RUVBL1/2, are ATPases associated with diverse cellular activities that form a complex with enhanced enzymatic activity through ring-shaped heterodimerization [1]. The RUVBL1/2 complex binds to chromatin and contributes to transcriptional regulation through chromatin remodeling [2,3]. For instance, RUVBL1 and RUVBL2 are subunits of ATP-dependent chromatin remodeling complexes such as INO80 and SWR1 [4]. The two proteins are also essential components of the TIP60 complex [5], which interact with oncogenic transcription factors such as cMyc [6] and E2F1 [7]. Increasing evidence support pathological functions of RUVBLs in a variety of cancers, including acute myeloid leukemia [8], breast cancer [9], colorectal cancer [10], head and neck cancers [11], hepatocellular carcinoma [12], gastric cancer [13], glioma [14], epithelial ovarian cancer [15], prostate tumors [16], renal cell carcinoma [17], and non-small cell lung carcinoma [18,19]. Depletion of RUVBLs suppresses cancer cell growth and progression [17,20,21,22,23].

Multiple myeloma (MM) is the second most common hematological cancer [24]. This disease of plasma cells progresses from monoclonal gammopathy of undetermined significance (MGUS), to smoldering myeloma, to newly diagnosed (ND) myeloma, to plasma cell leukemia (PCL). The standard of care for MM includes proteasome inhibitors such as bortezomib, immunomodulatory drugs like lenalidomide, and corticosteroids such as dexamethasone. New classes of drugs include monoclonal antibodies such as the CD38-targting daratumumab [25] and SLAMF7-targeting elotuzumab [26]. In addition, T-cell-engaging therapies, such as bispecific T-cell engagers [27] and chimeric antigen receptor T-cell therapies [28], emerged as groundbreaking approaches. Despite these advances, MM remains incurable due to the inevitable development of drug resistance, highlighting the needs to continuously seek for therapeutic strategies complementary to current standards of care.

CB-6644 is an allosteric small-molecule inhibitor that specifically targets the ATPase activity of the RUVBL1/2 complex [23]. This compound substantially reduces tumor burden without remarkable toxicity in xenograft mouse models of Burkitt lymphoma and MM, making it a promising pre-clinical drug to explore for MM treatment [23]. In this work, we characterized the transcriptome (gene expression) and regulome (chromatin accessibility) signatures of CB-6644 treatment in MM cells. We determined transcriptional pathways affected by CB-6644 through RNA-Seq data analysis and inferred the underling epigenetic regulatory mechanisms by analyzing chromatin accessibility. Integrative analysis of transcription responses initially identified CB-5083, an MM-suppressive p97 inhibitor [29], as a synergistic compound with CB-6644 in MM cells; however, subsequent experimental validation refuted this hypothesis.

## 2. Results

### 2.1. Clinical Relevance of RUVBL1 and RUVBL2 in MM

**Figure 1:**
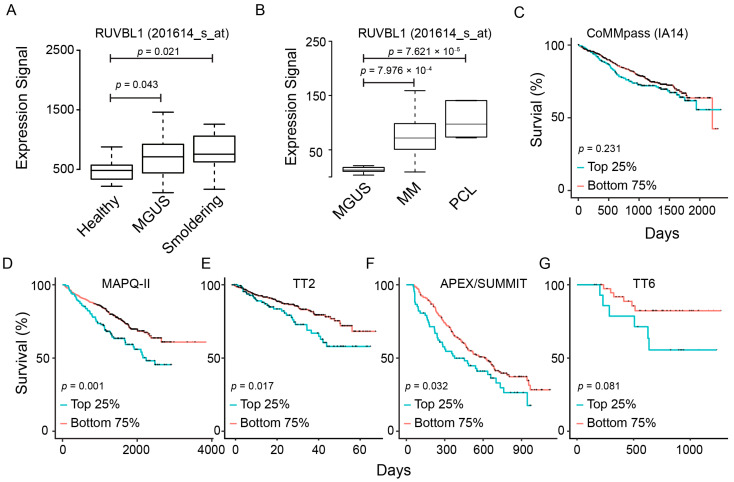
Clinical relevance of RUVBL1 expression in MM patients. (**A**) Comparison of RUVBL1 level between healthy donors and patients at disease stages of MGUS and smoldering. *p*-value by *t*-test. (**B**) Comparison of RUVBL1 level across patients at disease stages of MGUS, MM, and PCL. (**C**) K–M survival plot for RUVBL1 for ND MM patients from the CoMMpass trial. Patients sorted into the top 25% and others based on RUVBL1 expression (also applied to panels **D**–**G**). *p*-value by log-rank test. (**D**) K–M survival plot for RUVBL1 for ND MM patients from MAPQ-II (GEO: GSE24080); (**E**) K–M survival plot for RUVBL1 for ND MM patients from TT2 (GEO: GSE4204); (**F**) K–M survival plot for RUVBL1 for relapse patients from APEX/SUMMIT (GEO: GSE9782); (**G**) K–M survival plot for RUVBL1 for previously treated MM patients from TT6 (GEO: GSE57317).

To evaluate the clinical significance of RUVBL1/2 expression in MM, we compared their expression level among healthy donors and MM patients at different disease stages using http://www.genomicscape.com/ (accessed on 13 September 2022). In one gene expression dataset compiled by Zhan, et al. [30], RUVBL1 and RUVBL2 expression was substantially higher in plasma cells of patients with smoldering myeloma or MGUS than plasma cells from healthy donors (Figure 1A and Figure S1A). In another gene expression dataset compiled by Mattioli, et al. [31], we observed higher expression of RUVBLs in MM or PCL patients than in MGUS patients (Figure 1B and Figure S1B). Furthermore, we conducted a Kaplan–Meier (K–M) analysis on RUVBL1 and RUVBL2 as indicators of overall survival across several cohorts of MM patients. The initial analysis covered three cohorts of newly diagnosed (ND) MM patients: the CoMMpass clinic trial (IA14) [32], MAPQ-II [33], and TT2 [34]. The analysis revealed both RUVBL1 and RUVBL2 as unfavorable prognostic markers on overall survival (Figure 1C–E and Figure S1C–E). We extended the analysis to a cohort of MM patients who had previously received treatment (TT6) [35] and another cohort of relapse patients (APEX/SUMMIT) [36]. Consistent with our findings for ND MM patients, higher RUVBL1 or RUVBL2 expression predicted shorter overall survival for both previously treated MM patients and MM relapse patients (Figure 1F,G and Figure S1F,G). Therefore, an elevated expression of RUVBLs predicted worse MM disease progression.

### 2.2. CB-6644 Treatment Suppresses MM Growth and Induces Apoptosis

**Figure 2:**
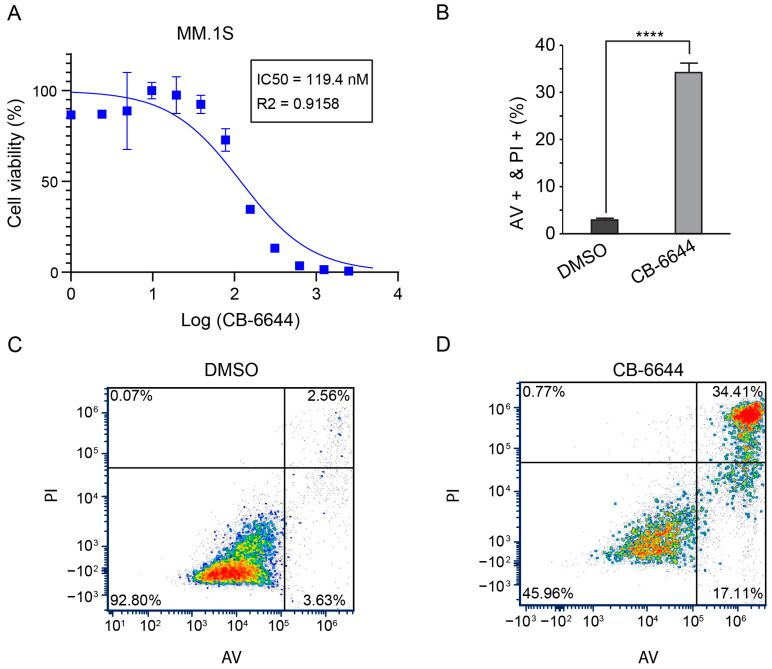
Proliferation suppression by CB-6644 in MM.1S. (**A**) IC50 of CB-6644 in MM.1S cells measured by CellTiter-Glo^®^ Luminescent Cell Viability Assay (*n* = 3). (**B**) Bar graph for the % of AV and PI double-positive cells of MM.1S following 72 h treatment with 120 nM CB-6644 (*n* = 3). ****: *p*-value < 0.0001 (*t*-test). (**C**,**D**) Representative flow panels of panel B. Live cells: lower left quadrant; apoptotic cells: lower right quadrant; dead cells: upper right quadrant. Red to blue means higher to lower density. AV: annexin V. PI: propidium iodide.

CB-6644 selectively inhibits the RUVBL1/2 complex by targeting ATPase activity at high potency [23]. To assess its effects on MM cells, we treated two MM cell lines (MM.1S and RPMI 8226) with CB-6644 for 72 h and determined the half maximal inhibitory concentration (IC50) using CellTiter-Glo^®^ Luminescent Cell Viability Assay: 120 nM for MM.1S and 60 nM for RPMI 8226 (Figure 2A and Figure S2A). For comparison, HS-5, a cell line for normal bone marrow stromal cells, showed an IC50 of 200 nM, thus resulting in less sensitivity to CB-6644 (Figure S2B). Similar to an observation involving melanoma cell lines [23], CB-6644 treatment induced substantial apoptosis in MM cells (Figure 2B and Figure S2C), with representative flow panels shown in Figure 2C,D and Figure S2D,E.

### 2.3. Transcriptome Signatures of CB-6644 Inhibition of RUVBL1/2 in MM Cells

**Figure 3:**
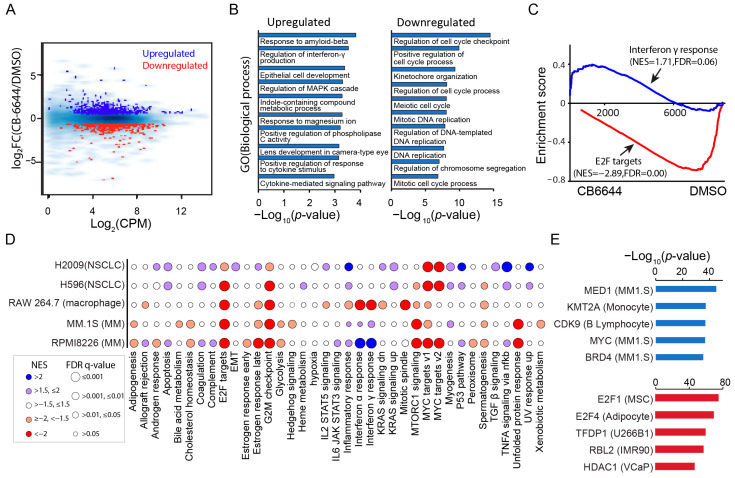
Molecular pathways affected by CB-6644 in MM cells. (**A**) MA plot displaying count per million (CPM; log_2_) and fold change (FC) of expression for the comparisons of CB-6644-treated cells (*n* = 2) vs. DMSO control cells (*n* = 3) in MM.1S. Blue: Genes upregulated in expression. Red: Downregulated genes. Light blue: All expressed genes. Shown for data generated in MM.1S, also applying to panels (**B**,**C**). (**B**) Gene ontology enrichment analysis on biological processes for the upregulated (**left** panel) or downregulated genes (**right** panel). (**C**) GSEA of expressed genes sorted by fold change in expression (CB-6644/DMSO) from high (left) to low (right) against MSigDB hallmark gene set “interferon γ response” (blue line) and “E2F targets” (red line). (**D**) Bubble plot visualization of results from GSEA enrichment analysis of CB-6644-induced expression changes in different cell systems (rows) against MSigDB hallmark gene sets (columns). Color indicates an overall upregulation (blue) or downregulation (red) of the gene set. Circle size indicates significance (FDR q-value). NES: normalized enrichment score. (**E**) Scatter plot visualization of the significance of transcriptional regulator inference from LISA for genes commonly upregulated (upper panel) or downregulated (lower panel) by CB-6644 in MM.1S and RPMI 8226. In parentheses are cells or tissues where the public ChIP-seq data are sourced from.

To determine the molecular pathways affected by CB-6644 in MM cells, we profiled the transcriptomes for two MM cell lines (MM.1S and RPMI 8226) treated with this inhibitor using RNA-Seq. CB-6644 treatment induced a substantial change in mRNA expression by upregulating 920 genes and downregulating 1110 genes in MM.1S (Figure 3A; Table S1). The treatment downregulated the mRNA expression of RUVBL1/2 by less than 5% (Figure S3A). Upregulated genes were enriched in biological processes related to interferon-γ production and MAPK cascade, while downregulated genes were enriched in biological processes related to cell proliferation (Figure 3B). Gene set enrichment analysis against hallmark gene sets of MSigDB obtained consistent results (Figure 3C).

We repeated the experiments for RPMI 8226. Like MM.1S, CB-6644 treatment modestly downregulated the mRNA expression of RUVBL1/2 (Figure S3A). Differential expressed (DE) gene analysis identified 2058 genes upregulated and 1289 downregulated by this inhibitor (Figure S3B; Table S2). Expression changes in DE genes were generally consistent between MM.1S and RPMI 8226 (Figure S3C). As expected, gene ontology enrichment analysis on biological processes revealed upregulation in interferon response and downregulation in cell proliferation (Figure S3D), further confirmed by Gene Set Enrichment Analysis (GSEA) on hallmark gene sets from MSigDB (Figure S3E).

Transcriptome signatures of RUVBL1/2 inhibition have recently been explored in other cell systems. Assimon et al. [23] examined the cellular consequence of RUVBL1/2 inhibition in melanoma cells when they first introduced CB-6644. Yenerall et al. [37] discovered a structurally similar molecule for RUVBL1/2 inhibition (known as compound B) and assessed its transcriptomic effects in two non-small cell lung cancer cells (NSCLCs). Zhang et al. [38] investigated the impact of CB-6644 inhibition on inflammatory response in a macrophage-like cell line. We downloaded their expression data if publicly available [37,38] and conducted a comparative analysis on their transcriptome signatures with this study. The analysis revealed that gene sets related to cell proliferation such as E2F targets and G2M checkpoint were commonly downregulated across all datasets (Figure 3D). In contrast, upregulation of interferon-responsive genes was MM.1S- and RPMI 8226-specific, with a downregulation and no substantial change observed in macrophage (RAW 264.7) and NSCLC (H2009 and H596), respectively (Figure 3D).

To understand the regulatory mechanisms underlying the transcriptional response to CB-6644, we applied LISA, which uses TF target genes defined from public ChIP-seq and chromatin accessibility data to predict transcriptional regulators [39]. For genes commonly downregulated by CB-6644 in MM.1S and RPMI 8226, E2F1 and E2F4 emerged as the top transcription factor (Figure 3E). Transcription regulators predicted for genes commonly upregulated included MED1, MYC, CDK9, and a H3K4me3 methyltransferase KMT2A (Figure 3E).

### 2.4. Regulome Signatures of CB-6644 Inhibition of RUVBL1/2 in MM Cells

**Figure 4:**
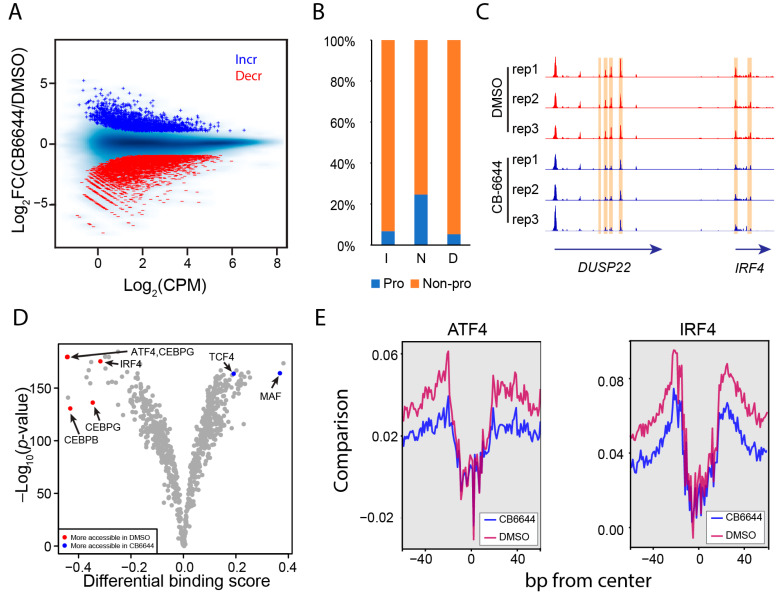
CB-6644 induced transformation of accessible chromatin in MM cells. (**A**) MA plots displaying CPM (log_2_) and FC of chromatin accessibility for CB-6644 treated (*n* = 3) vs. DMSO control (*n* = 3) MM cells. Blue: chromatin-accessible regions increasing in accessibility (“Incr”); red: chromatin-accessible regions decreasing in accessibility (“Decr”); light blue: all chromatin-accessible regions. (**B**) Distribution in promoter (transcription start sites ±2500 bps; Pro) and non-promoter regions (“Non-pro”) for chromatin-accessible regions sorted by their changes in response to CB-6644 treatment: “I” for increasing, “D” for decreasing, and “N” for no change. (**C**) IGV genome browser image showing the distribution of Omni-ATAC read density across a genomic region enclosing *DUSP22* and *IRF4* for samples treated with CB-6644 (blue) or DMSO (red). *Y*-axis normalized by total library size and adjusted to the same scales. Highlighted in yellow are genomic regions showing a decrease in chromatin accessibility. (**D**) Volcano plots for differential binding activity vs. the -log_10_ (*p*-value) for all TF motifs (dots) from JASPAR. Highlighted DMSO-specific TFs are labeled in red, while CB-6644-specific factors are in blue. (**E**) Bias-corrected Tn5 signals indicating chromatin accessibility centered on motifs corresponding to ATF4::CEBP (**left** panel) and IRF4 binding (**right** panel) for CB-6644-treated cells and DMSO control cells.

Since RUVBL1/2 are subunits of several chromatin remolding complexes [4], chromatin reorganization could be one source of regulatory mechanisms underlying the dramatic transcription response to CB-6644. To this end, we profiled chromatin accessibility for MM.1S cells treated with CB-6644 by using Omni-ATAC [40]. The analysis identified ~80,000 reproducible open chromatin regions. Differential accessible region (DAR) analysis revealed that chromatin became generally more compact after the treatment: 4486 regions decreased vs. 1995 regions increased in chromatin accessibility (Figure 4A). Changes in accessibility occurred preferentially beyond promoter regions (Figure 4B). This was exemplified by a 125k-bp genomic region enclosing *DUSP22* and *IRF4* (Figure 4C). We annotated potential target genes for DARs using GREAT [41] and observed concordant changes in chromatin accessibility and mRNA expression at their target genes: An increase in chromatin accessibility predicted expression upregulation, while a decrease predicted expression downregulation (Figure S4A). Genes downregulated and targeted by decreased DARs signified biological processes linked to the regulation of transporter activity, regulation of replication, and response to extracellular stimulus (Figure S4B).

To identify transcriptional regulators involved in the chromatin reorganization in response to CB-6644, we applied footprinting analysis to the Omni-ATAC data for TF binding dynamics [42]. The analysis identified a decrease in TF binding level for the ATF4/CEBP heterodimer and IRF4 (Figure 4D). Moreover, the decrease in binding level was accompanied by a chromatin compaction at their binding sites (Figure 4E), indicating a positive role of ATF4/CEBP and IRF4 in promoting chromatin accessibility. For TFs that increased in genomic binding level, footprint analysis identified MAF and TCF4 (Figure 4D). However, the increase in TF binding level for MAF and TCF4 was not accompanied by an increase in chromatin accessibility (Figure S4C). Lastly, we employed TF binding site enrichment analysis using public ChIP-seq data collected from ChIP-Atlas [43] to identify TFs enriched in the DARs. Consistent with the footprinting analysis, DARs decreased in accessibility were enriched in TF binding of ATF4, CEBPβ, and IRF4, while DARs increased in accessibility were enriched in TCF4 binding sites (Figure S4D).

### 2.5. Molecular Signatures of Genes Exhibiting Reduced Accessibility at Multiple Regulatory Sites

**Figure 5:**
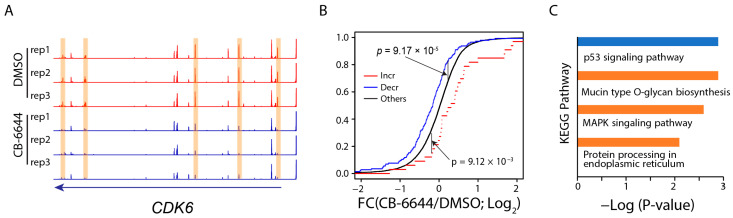
Genes associated with a decrease in chromatin accessibility at multiple regulatory regions. (**A**) IGV genome browser image showing the distribution of Omni-ATAC read density across a genomic region enclosing CDK6 for samples treated with CB-6644 (blue) or DMSO control (red). Highlighted are genomic regions showing a decrease in chromatin accessibility. (**B**) Empirical cumulative distribution of the expression FC (CB-6644/DMSO) of genes associated with multiple genomic regions that increased (red) or decreased (blue) in chromatin accessibility. Black: all expressed genes. A line shifting to the right indicates an overall increase in expression. *p*-value by the Kolmogorov–Smirnov (K-S) test. (**C**) KEGG pathway enrichment analysis for genes associated with multiple genomic regions that increased (blue) or decreased (brown) in chromatin accessibility.

The prediction of gene targets for DARs through GREAT analysis [41] identified a handful of expressed genes that increased (*n* = 33) or decreased (*n* = 144) chromatin accessibility at multiple regulatory regions in response to the CB-6644 treatment (see *CDK6* as an example; Figure 5A); Tables S3 and S4 contain full lists of the genes and their associated peak regions. As expected, accessibility decreasing at multiple regions predicted expression downregulation of the associated genes, while accessibility increasing predicted expression upregulation (Figure 5B). Downstream functional inference revealed that the 33 genes were enriched in the p53 signaling pathway, while the 144 genes were enriched in O-glycan biosynthesis, MAPK signaling pathway, and protein processing in the endoplasmic reticulum (Figure 5C).

### 2.6. Chemical Compounds Synergistic with CB-6644 Inferred by Transcriptome Analysis

**Figure 6:**
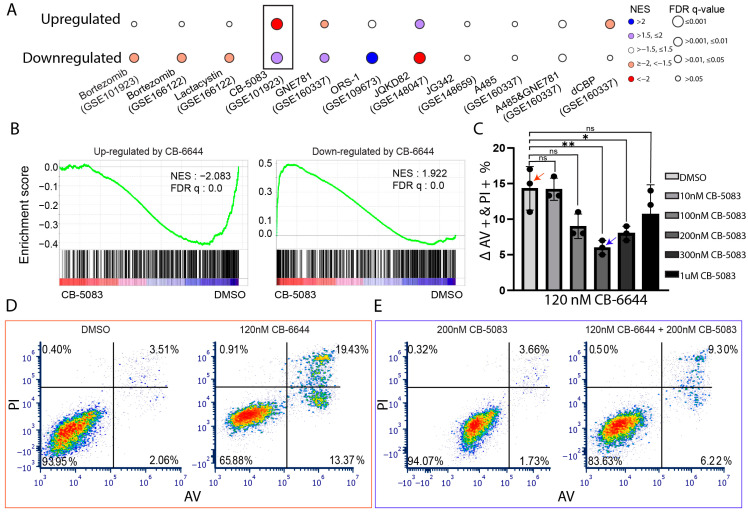
Inference of synergistic compounds with CB-6644 through integrative gene expression analysis. (**A**) Bubble plot visualization of results from GSEA enrichment analysis of expression changes induced by various chemical compounds in MM cells, as collected from GEO with GSE# indicated in parentheses, against gene sets (rows) that are commonly upregulated or downregulated by CB-6644 in MM.1S and RPMI 8226. Color indicates expression upregulation (blue) or downregulation (red) of the gene sets by the indicated compounds (columns). Circle size indicates FDR q-value. Box: GSEA results detailed in panel B. (**B**) GSEA of expressed genes sorted by expression FC in response to CB-5083 from high (red spectrum) to low (blue spectrum) against gene sets downregulated or upregulated by CB-6644. (**C**) % of PI and AV double-positive cells from treatment with 120 nM CB-6644 combined with varying concentrations of CB-5083 in MM.1S cells for 72 h, relative to the basal effect of CB-5083 alone or DMSO alone. *: *p* < 0.05. ** *p* < 0.005 (*t*-test). ns: not significant. *n* = 3 for each condition. Red and blue arrows correspond to the examples shown in panels (**D**,**E**), respectively. (**D**) Representative flow panel for the % of PI and AV double-positive cells, indicated as the red arrowhead in panel **C** from CB-6644 treatment (120 nM) relative to DMSO alone (19.43–3.51%). Red to blue means higher to lower density. (**E**) Representative flow panel for the % of PI and AV double-positive cells, indicated as the blue arrowhead in panel **C** from the combined treatment of CB-6644 (120 nM) and CB-5083 (200 nM) relative to CB-5083 alone (200 nM) (9.30–3.66%). Live cells: lower left quadrant; apoptotic cells: lower right quadrant; dead cells: upper right quadrant.

Transcriptome signatures represent an overall summary of a chemical compound’s effect on the cellular state of a cell population [44]. A comparison of the signatures among chemical compounds in the same cells helps to identify potential synergistic drugs. A negative correlation predicts a compound to revert the transcriptome to a state that is sensitive to the other compound [44]. To this end, we collected RNA-Seq data deposited in GEO for a variety of compound treatments in MM cells and compared their expression changes to those induced by CB-6644 (Figure 6A). We focused on genes upregulated or downregulated by CB-6644 (shared by MM.1S and RPMI 8226) and examined expression changes induced by other compounds. GSEA analysis identified the p97 inhibitor CB-5083 [29] as a potential drug to be synergistic with CB-6644: Expression changes induced by CB-6644 and CB-5083 were generally opposite (Figure 6B and black box in Figure 6A). Leading genes from the GSEA analysis for those downregulated by CB-6644 but upregulated by CB-5083 were enriched in the biological process related to the biosynthesis of amino acids, while those upregulated by CB-6644 but downregulated by CB-5083 were related to inflammatory response (Figure S5A).

We experimentally tested the potential drug synergy between CB-6644 and CB-5083. Briefly, we treated MM.1S cells with 120 nM CB-6644 in combination with various concentrations of CB-5083 and after 72 h measured % of PI and annexin V double-positive cells, which indicate dead cells. The results revealed that CB-6644 and CB-5083 were generally antagonistic rather than being synergistic (Figure 6C–E). Similar antagonistic effects between the two compounds were observed for RPMI-8226 (Figure S5B–D).

## 3. Discussion

RUVBL1/2 are essential proteins implicated in many biological processes, such as transcriptional regulation [45], chromatin remodeling [3], energy and glucose/lipid metabolism [46,47], Pol II clustering in nucleus [48], DNA replication [37], and DNA repair [49]. Pathogenetic roles of RUVBL1/2 in diseases may include inflammatory response [38], cancer invasion/metastasis [9,19,50,51], drug resistance [16], and radio resistance [37]. Despite the significance in oncogenesis [52], to our knowledge, the function of RUVBL1/2 in MM remains unexplored. Our analysis of public expression data from MM patients revealed a positive correlation of RUVBL1/2 expression with MM disease progression. We identified both genes as unfavorable prognosis markers for both ND and relapsed MM patients. Therefore, the RUVBL1/2 complex is a worthy target for the development of novel therapeutic strategies of MM treatment.

CB-6644 is a selective inhibitor of the RUVBL1/2 complex with anticancer activity [23]. This compound reduces ATPase activity of RUVBL1/2 complex by 50% but exerts over 95% of cell killing. The promise of CB-6644 as a therapeutic agent stems from its antitumor activity without obvious toxicity, as demonstrated in xenograft models of Burkitt’s lymphoma [23], Ewing sarcoma [53], multiple myeloma [23], and prostate cancers [16], as well as orthotopic transplant models of pancreatic ductal adenocarcinoma [54]. Intriguingly, untransformed, normal human fibroblasts are less sensitive to CB-6644 than cell lines representative of colon, lung, and pancreatic cancers [55]. We made a similar observation for HS-5, a cell line representing normal bone marrow stromal cells, when compared to MM cell lines. Nevertheless, the modest effect of CB-6644 on normal cells suggests the need for future work to broaden its therapeutic window, achieving the desired effects without unacceptable toxicities while customizing to specific cancers [37].

The high efficacy of CB-6644 in antitumor activity in the MM xenograft mouse model [23] makes it an attractive chemical probe to understand RUVBL biology in this plasma cell cancer. As a first step to this end, we defined molecular signatures of MM cells in response to CB-6644 treatment using genome-wide profiling techniques such as RNA-Seq for transcriptome and Omni-ATAC for regulome.

Transcriptional response to RUVBL1/2 inhibition has been a topic of recent studies in other cell systems, such as NSCLCs and macrophages [37,38]. Our comparative analysis to the signatures defined for MM cells identified common molecular pathways downregulated by CB-6644: E2F targets, G2M checkpoints, and Myc targets, all of which regulate cell proliferation. Indeed, chemical inhibition of ATPase activities of the RUVBL1/2 complex suppresses DNA replication [37] and induces cell cycle arrest [23]. The most upregulated pathways in MM cells were related to inflammatory response and interferon response, whereas in macrophages, CB-6644 suppressed inflammatory response [38]. Upregulation of the stress-responsive TP53 pathway as reported in melanoma A375 cells [23] also occurred in NSCLC cells [37] and MM cells but not in macrophage [38]. Therefore, while CB-6644 inhibition of RUVBL1/2 universally suppresses cell proliferation across different cell systems, transcription pathways activated by this inhibitor are cell-type specific.

Transcriptional regulator inference analysis for genes downregulated by CB-6644 in MM cells identified E2F1 as one of the top candidates. In MM cells, E2F1 predominately occupies the promoter of active genes involved in cell proliferation [56]. This transcription factor physically interacts with the RUVBL1/2 complex, as supported by co-immunoprecipitation experiments [7,14]. As for transcriptional regulation, E2F1 recruits RUVBL1/2 to chromatin as co-activators to amplify expression of E2F1 targets in a manner depending on the ATPase activity of RUVBL1/2 [7,14,57]. Knockdown of E2F1 in MM cells induces cell cycle arrest and subsequent apoptosis [56]. From our work, CB-6644 treatment downregulated the mRNA expression level of E2F1 in MM cells. Therefore, the CB-6644-induced downregulation of E2F1 targets in MM cells may stem from a combinatory effect of a sub-optimal recruitment of RUVBL1/2 by reducing E2F1 expression and a compromised transcription amplification by reducing the ATPase activity of RUVBL1/2.

RUVBL1/2 are essential components of several chromatin remodeling complexes, such as the INO80 complex for nucleosome sliding and H2A/H2B histone exchange, the TIP60 complex for histone acetylation, and the Swr1/SRCAP complex for histone variant exchanges [3]. Dysregulation of RUVBL1/2 expression and their ATPase activity may regulate transcription response through modulating chromatin. Knockdown of RUVBL2 mRNA expression level reduces chromatin accessibility at promoters of E2F1 target genes and limits transcription amplification [7]. Inhibition of ATPase activity of RUVBL1/2 in macrophages reduces H3K4 trimethylation at promoters of pro-inflammatory genes and compromises their transcription activation during inflammatory response [38]. In MM cells, CB-6644 inhibition of ATPase activity of RUVBL1/2 induced a global chromatin compaction at distal regulatory regions, with concordant changes in the expression of their target genes.

Mechanisms on how RUVBL1/2 promote chromatin accessibility at distal regulatory regions could be through TFs. Prominent candidates identified from our analysis included ATF4::C/EBP and IRF4, of which their predicted binding sites were enriched at regulatory regions that exhibited a decrease in chromatin accessibility induced by CB-6644. The RUVBL1/2 complex interacts with C/EBPα and C/EBPβ through tandem affinity purification and mass spectrometry analysis [58]. Both ATF4::CEBP and IRF4 are implicated as pioneer transcription factors promoting chromatin accessibility at their target sites [59,60,61,62,63,64]. Other mechanisms may include the facilitation of histone acetylation by the TIP60 complex and the exchange of histone variant H2A.Z by the SRCAP complex, both markers known to associate with active chromatin configurations at enhancers for transcription activation [65,66,67].

As an effort to identify potential synergistic compounds of CB-6644, we computationally screened multiple chemical compounds tested in MM cells through comparative analysis of transcriptional responses. The analysis identified CB-5083, which induces an expression change in a manner opposite to CB-6644. However, experimental validation identified the two compounds as being antagonistic rather than synergistic. Interestingly, both compounds interfere with separate but essential processes that maintain cellular homeostasis [29]. It is possible that disrupting one pathway with CB-5083 (protein degradation) could activate compensatory responses that reduce the impact of inhibiting the other pathway with CB-6644 (chromatin remodeling).

Lastly, it is important to acknowledge the potential limitations related to the choice of cell lines in our study. While MM.1S and RPMI 8226 are among the most cited cell lines in MM research, they do not represent the most observed genomic translocation events in patients, such as those involving IgH and CCND or WHSC1 [68,69,70]. However, MM.1S is considered one of the most patient-relevant cell lines in terms of transcriptomic similarity [71]. Additionally, the reliability of our findings is reinforced by the consistent transcriptome signature observed in both MM.1S and RPMI 8226, which is distinctive from other non-MM cell lines [37,38]. Future studies could further substantiate these findings by including additional MM cell lines, such as ANBL-6, which is highly rated for mimicking patient expression profiles [71].

## 4. Materials and Methods

### 4.1. Cell Lines

MM cell lines MM.1S (ATCC, CRL-2974) and RPMI 8226 (ATCC, CCL-155), and the BM stromal cell line HS-5 (ATCC, CRL-11882), were obtained from the American Type Culture Collection (Manassas, VA, USA). These cell lines are tested for mycoplasma every six months. The cells were maintained in RPMI-1640 (ATCC, 30-2001), supplemented with 10% Fetal Bovine Serum (FBS) (Gibco, Waltham, MA, USA, 10082-147) and 1% penicillin/streptomycin (Gibco, Waltham, MA, USA, 15140122). For growth, the cells were kept in a Heracell™ VIOS 160i CO_2_ Incubator with 5% CO_2_ at 37 °C.

### 4.2. IC50 Assays and Apoptosis Assays

MM.1S and RPMI 8226 suspension cells were plated at a density of 10,000 cells per well in 96-well plates. Cells were treated with CB-6644 (MedChemExpress, Monmouth Junction, NJ, USA, HY-114429) at concentrations ranging from 1 nM to 5 µM to assess its impact on cell viability over a 72 h treatment period, all in triplicate. Following this, cell viability was evaluated using the CellTiter-Glo^®^ Luminescent Cell Viability Assay (Promega Corporate, Madison, WI, USA, G7571), following the manufacturer’s instructions. Luminescence, which reflects ATP content and thus the number of viable cells, was measured using a BioTek Synergy HTX multi-mode reader (Agilent, Santa Clara, CA, USA). Luminescent data were normalized to control wells, and IC50 values for CB-6644 were calculated using non-linear regression analysis by plotting the range of concentrations against the response.

To measure apoptosis, 2 × 10^5^ MM.1S or RPMI 8226 cells were plated onto 6-well plates and treated with 120 nM or 60 nm CB-6644 for 72 h (IC50), respectively, all in triplicate. Following this, the cells were harvested and stained for flow cytometry using the Annexin V Apoptosis Detection Kit with PI (Biolegend, San Diego, CA, USA, 640914, 640932) following the manufacturer’s protocol. Samples were run on LSR Fortessa and analyzed using FACSDiva™ Software v8.0 (BD biosciences, Franklin Lakes, NJ, USA).

### 4.3. Synergy Assay

MM.1S and RPMI 8226 cell lines were plated at a density of 10,000 cells per well in complete RPMI 1640 media. MM.1S cells were treated with 120 nM CB-6644 (IC50) and incubated for 72 h. As RPMI 8226 is more sensitive to CB-5083 [29], the cells were treated with 30 nM CB-6644 (half of IC50) and incubated for a short period of 48 h. This design was to leave enough space to observe its potential synergy with CB-5083 (MedChemexpress, HY-12861) in cell killing (measured by % dead cells) as CB-5083′s concentration varied. Following incubation, the cells were stained using the BioLegend Apoptosis Detection Kit (Biolegend, 640932) and analyzed on a Cytek Aurora using SpectroFlo software v3.03 (Cytek Biosciences, Fremont, CA, USA).

### 4.4. FACS Isolation of Live Cells Following CB-6644 Treatment for Omni-ATAC and RNA-Seq

Omni-ATAC assay for chromatin accessibility requires live cells [40], which were isolated through FACS as follows: MM.1S and RPMI 8226 cells were plated at a density of 500,000 cells per well in 6-well plates, each containing 2 mL of complete RPMI-1640 media. MM.1S cells were incubated with 120 nM CB-6644 (IC50), while RPMI 8226 cells were incubated with a reduced concentration at 40 nM instead of the IC50 (60 nm), to increase the yield of live cells from FACS isolation. Both cell lines were treated for 72 h. Following incubation, the cells were stained with live/dead stain (Invitrogen, Waltham, MA, USA, L10119A). The cells were excited using a 628 nm red laser and detected using a detector with a 780/60 bandpass filter. The stained cells were then sorted for live cells (L10119A negative) using the FACAria III flow cytometer (BD Bioscience, 648282) and analyzed by the FACS Diva software 9.0 (BD Bioscience). The isolated cells were separated into half for Omni-ATAC and another half for RNA-Seq.

### 4.5. RNA-Seq and Omni-ATAC 

Total RNA for each sample was extracted using the Qiagen RNeasy Plus Mini Kit (Qiagen, Hilden, Germany, 74134). RNA-Seq libraries were prepared by Admera Health (South Plainfield, NJ, USA) using NEBNext ultra II RNA (Directional) with polyA selection. Omni-ATAC libraries [40] for chromatin accessibility were prepared using a Tagment DNA Enzyme and Buffer Large Kit (Illumina, San Diego, CA, USA, 20034198) and following procedures described previously [61]. Specifically, 50,000 live cells from flow sorting per replicate were collected and cells were lysed using ATAC-Resuspension Buffer (RSB) (10 mM Tris-HCl pH 7.4, 10 mM NaCl, 3 mM MgCl_2_) containing 0.1% NP40 (Thermo Scientific, Waltham, MA, USA, 85124), 0.1% Tween-20 (Fisher Scientific, Waltham, MA, USA, BP337-500), and 0.01% Digitonin (Promega, Madison, WI, USA, G9441). The lysis was removed through several washes with ATAC-RSB containing 0.1% Tween-20 but not NP40 or digitonin. The nuclei were then pelleted by centrifugation at 500 RCF for 10 min at 4 °C. The nucleus pellet was then incubated in a 50 µL transposase mix (25 µL of 2× TD buffer, 2.5 µL of transposase, 16.5 µL of PBS, 0.5 µL of digitonin, 0.5 µL Tween 20, and 5 µL of nuclease free water) in a thermomixer at 37 °C, 1000 rpm for 30 min. Following this, the DNA was purified with a DNA Clean and Concentrator-5 Kit (Zymo, Irvine, CA, USA, D4014). The purified DNA sample was amplified by 12 cycles of PCR using primers and temperatures outlined in Buenrostro et al. [72] using NEB Next Master mix (New England Biolabs, Ipswich, MA, USA, M0541S). The DNA was then yet again purified using the Zymo DNA clean and concentrator. Following this, the ATAC libraries were run on a 2% agarose gel. The nucleosome pattern and size were observed, and the gel was cut ~200–600 bp and the DNA was purified from the gel using the Qiagen gel MinElute Gel Extraction Kit (Qiagen, Hilden, Germany, 28606). RNA-Seq and Omni-ATAC libraries were sequenced by Admera Health (South Plainfield, NJ, USA) using Illumina Nova-Seq 6000 and HiSeq 2500 (2 × 150 pair-end), respectively.

### 4.6. Data Analysis

RNA-Seq data analysis followed our previous works [61,73,74]: RNA-Seq read alignment to the human genome (hg38) by subread v2.0.1 [75], summarization of read counts to transcripts at the gene level by featureCounts [76], RPKM qualification [77] of mRNA expression level by an in-house script, call of differentially expressed genes by EdgeR3 (FDR < 0.05 and fold change > 1.5) [78], functional enrichment analysis against hallmark gene sets from MSigDB [79] by GSEA [80], gene ontology or pathway enrichment analysis by MetaScape [81], and transcriptional regulator inference by LISA [39] or TFEA.ChIP [82].

Omni-ATAC sequencing data analysis followed our previous work [61]: read alignment to hg38 by Bowtie2 [83], which is based on the Burrows–Wheeler Transform [84]; visualization of reads distribution across gene locus by IGV [85]; call peaks by MACS3 [86]; prediction of differentially accessible regions (DARs) by EdgeR3 (FDR < 0.01 and fold change > 2) [78]; target gene predictions for DARs by GREAT [41]; footprinting analysis by TOBIAS [42]; and TF enrichment analysis by ChIP-Atlas [43].

## 5. Conclusions

In conclusion, our study underscores the potential of CB-6644, a selective inhibitor of the RUVBL1/2 complex, as a promising anti-tumor agent in MM. CB-6644 demonstrated significant anti-tumor activity by suppressing cell proliferation and inducing apoptosis in MM cells. Through transcriptome and regulome analyses, we identified key molecular pathways responsive to CB-6644 treatment, as well as transcription factors involved in regulating the transcriptional response via modulation of chromatin accessibility. The observed antagonistic interaction with CB-5083, a known MM-suppressive p97 inhibitor, highlights the need for further research to fully understand the benefits and limitations of RUVBL1/2 inhibition in developing more effective therapies for this incurable plasma disease.

## Data Availability

ATAC-seq and RNA-Seq sequencing data were deposited to the Gene Expression Omnibus with accession # GSE271838 and GSE271840, respectively.

## Supplementary Materials

The following supporting information can be downloaded at: https://www.mdpi.com/article/10.3390/ijms25169022/s1.
