# Supplementary material for: Molecular Signatures of CB-6644 Inhibition of the RUVBL1/2 Complex in Multiple Myeloma
Source: Int J Mol Sci. 2024 Aug 20;25(16):9022. doi: 10.3390/ijms25169022 (PMC11354775; doi:10.3390/ijms25169022)
Supplement: Supplementary file 1 [file ijms-25-09022-s001.zip › Supplementary Figures-rv-08192024.pdf]

Supplemental Figures

Molecular Signatures of CB-6644 Inhibition of the RUVBL1/2 Complex in Multiple Myeloma

Weijun Yi†, Sebastian A. Dziadowicz†, Rachel S. Mangano, Lei Wang, Joseph McBee, Steven M. Frisch, Lori A. Hazlehurst, Donald A. Adjeroh*, Gangqing Hu*

†: co-first authors; *: co-corresponding authors

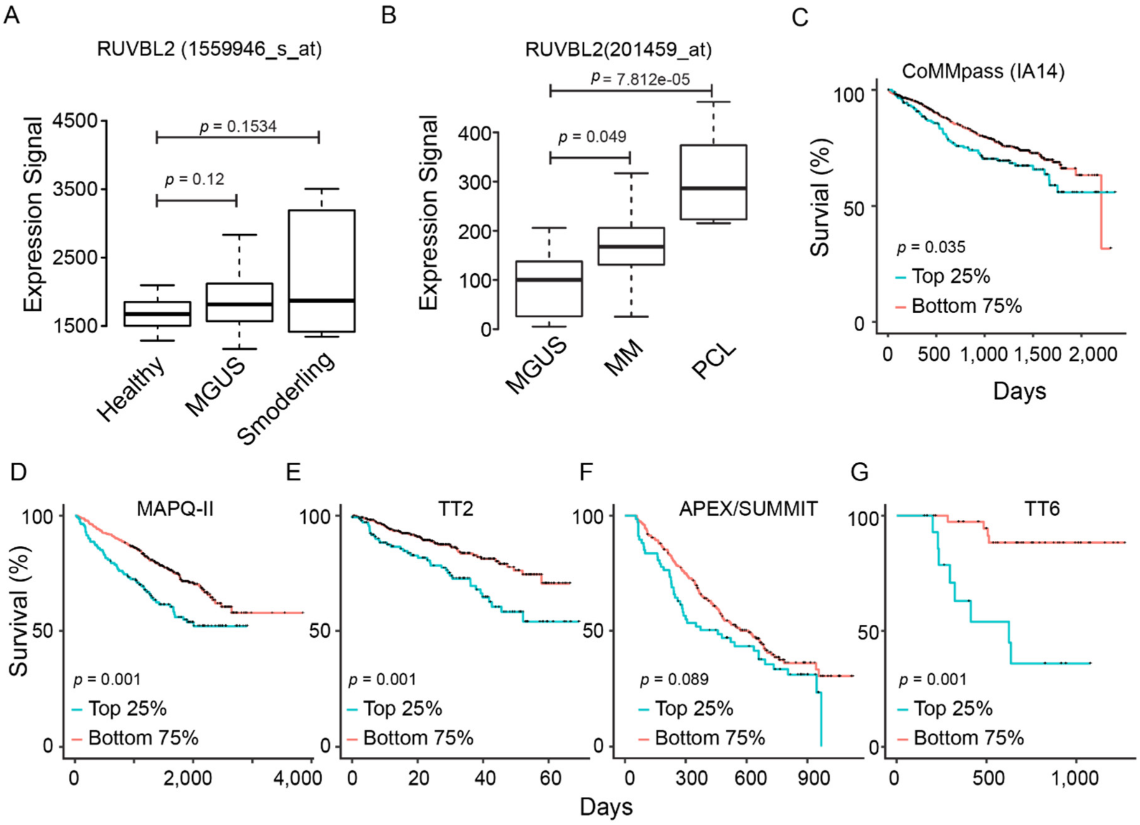

Figure S1: Clinical relevance of RUVBL2 expression in MM patients. **(A)** Comparison of RUVBL2 level between healthy donors and patients at disease stages of MGUS and smoldering. P-value by t-test. (**B**) Comparison of RUVBL2 level across patients at disease stages of MGUS, MM and PCL. P-value by t-test. (**C**) K–M survival plot for RUVBL2 for ND MM patients from the CoMMpass trial. Patients sorted into the top 25% and others based on RUVBL2 expression (also applied to panels **D** to **G**). p-value by log-rank test. (**D**) K–M survival plot for RUVBL2 for ND MM patients from MAPQ-II (GEO: GSE24080). (**E**) K–M survival plot for RUVBL2 for ND MM patients from TT2 (GEO: GSE4204). (**F**) K–M survival plot for RUVBL2 for relapse patients from APEX/SUMMIT (GEO: GSE9782). (**G**) K–M survival plot for RUVBL2 for previously treated MM patients from TT6 (GEO: GSE57317).

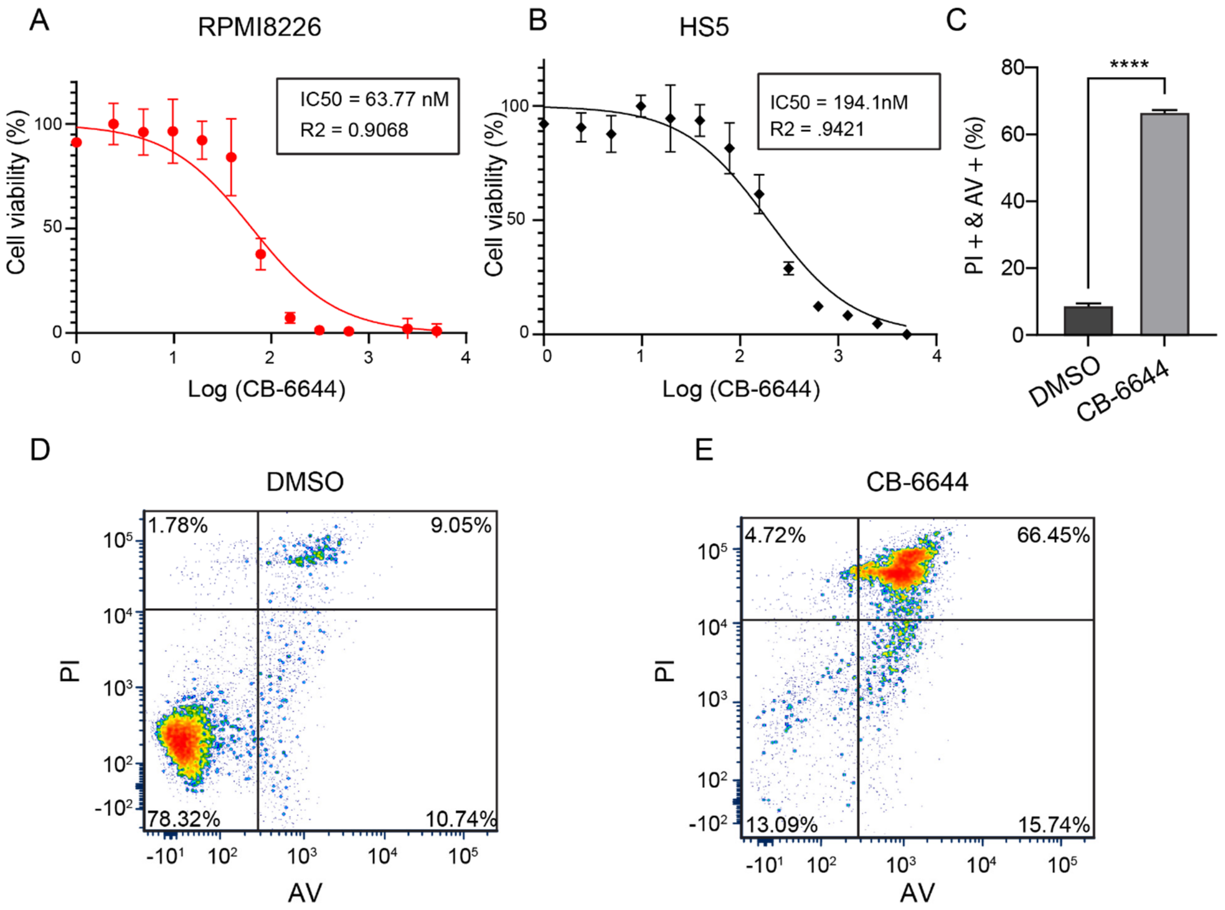

Figure S2: Proliferation suppression by CB-6644 in RPMI 8226. (**A**) IC50 of CB-6644 in RPMI 8226 cells measured by CellTiter-Glo® Luminescent Cell Viability Assay (n=3). (**B**) IC50 of HS-5 (n=3). (**C**) Bar graph for the % of both Annexin V and PI (double positive) statistical analysis done via unpaired T-test (**** $P < .0001$) of RPMI 8226 following 72 hours treatment with 60nM CB-6644 (n=3). (**D-E**) Representative flow panels of panel **B**. PI: Propidium Iodide. Live cells: lower left quadrant; apoptotic cells: lower right quadrant; dead cells: upper right quadrant.

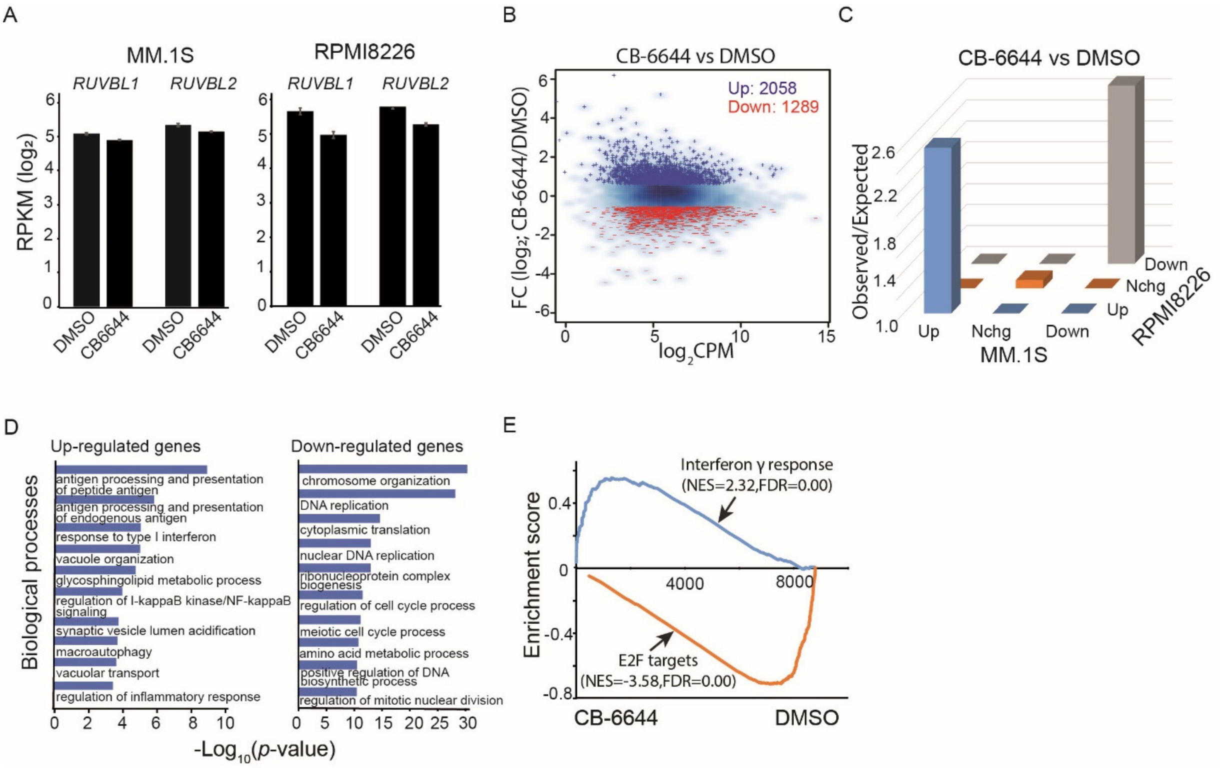

Figure S3: Molecular pathways affected by CB-6644 in RPMI 8226. (A) CB-6644-induced expression changes for RUVBL1 and RUVBL2 in MM.1S and RPMI 8226 cells. (**B**) MA-plot displaying CPM (log$_2$) and expression FC (log$_2$) for CB-6644 treated cells (n=3) vs. DMSO control cells (n=3) in RPMI 8226. Red: Genes downregulated in expression. Blue: Genes upregulated. Light blue: All expressed genes. (**C**) Bar plot for observed versus expected number of genes (z-axis), sorted based on transcription responses to CB-6644 in MM.1S cells (x-axis) and in RPMI 8226 cells (y-axis). "Up": up-regulated in expression; "Down": Down-regulated; "Nchg": No expression change. (**D**) Gene ontology enrichment analysis on biological processes for genes upregulated (left panel) or downregulated (right panel) by CB-6644 in RPMI 8226 cells. (**E**) GSEA of expressed genes sorted by expression FC (CB-6644/DMSO) from high (left) to low (right) in RPMI 8226 cells against MSigDB hallmark gene set "interferon gamma response" (blue line) and "E2F targets" (brown line).

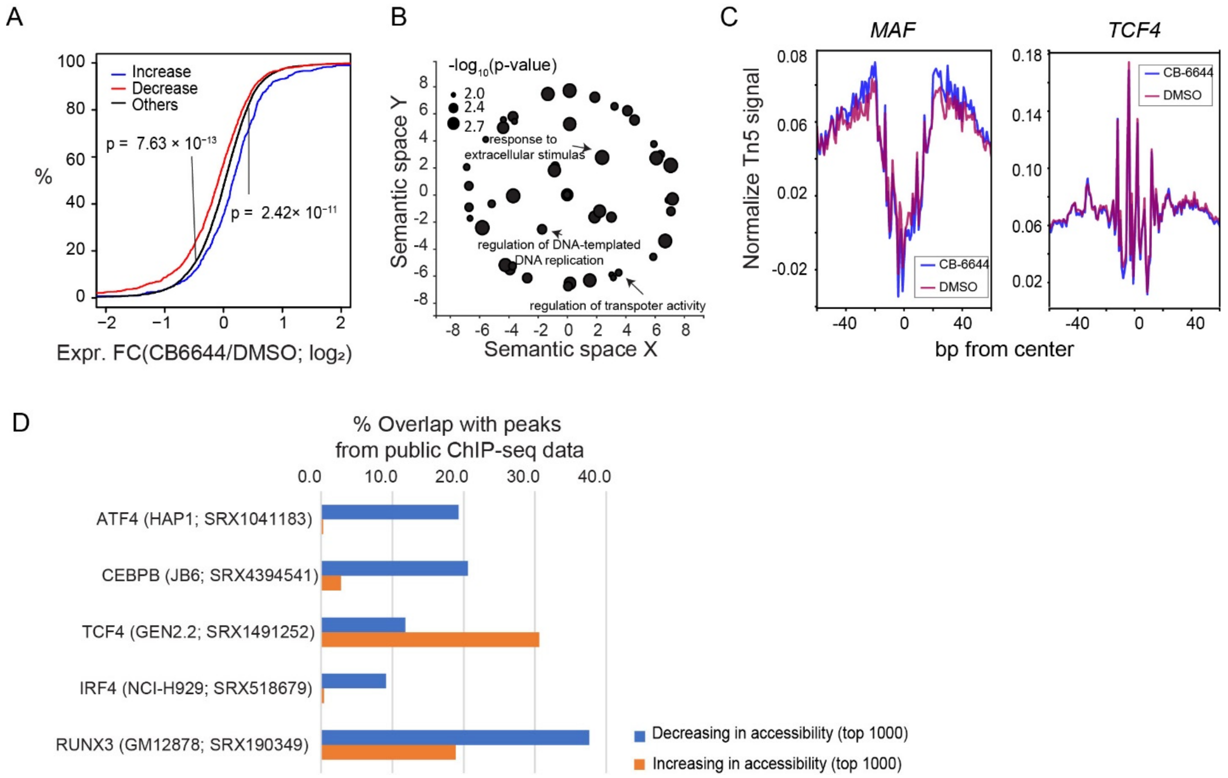

Figure S4: Transcription factor inference for DARs with public ChIP-seq data. (**A**) Empirical cumulative distribution profile for expression FC (CB-6644/DMSO) of target genes predicted for peaks showing increase (blue), decrease (red), or no change (black) in accessibility by CB-6644. A line shifting to the right indicates an overall increase in expression. p-value by the Kolmogorov–Smirnov (K-S) test. (**B**) Visualization of enriched gene ontology terms (depicted as circles) for genes downregulated and targeted by genomic regions decreased in chromatin accessibility. Color and size of circles indicate significance of enrichment $\log_{10}$ p value. (**C**) Bias corrected Tn5 signals for chromatin accessibility centered on motifs corresponding to MAF (left panel) and TCF4 binding (right panel) for CB-6644-treated cells and DMSO control cells. (**D**) Bar plots for the % of genomic regions containing TF binding sites predicted from public ChIP-Seq data for the top 1,000 regions that decreased in chromatin accessibility and for the top 1,000 regions that increased in chromatin accessibility in response to CB-6644.

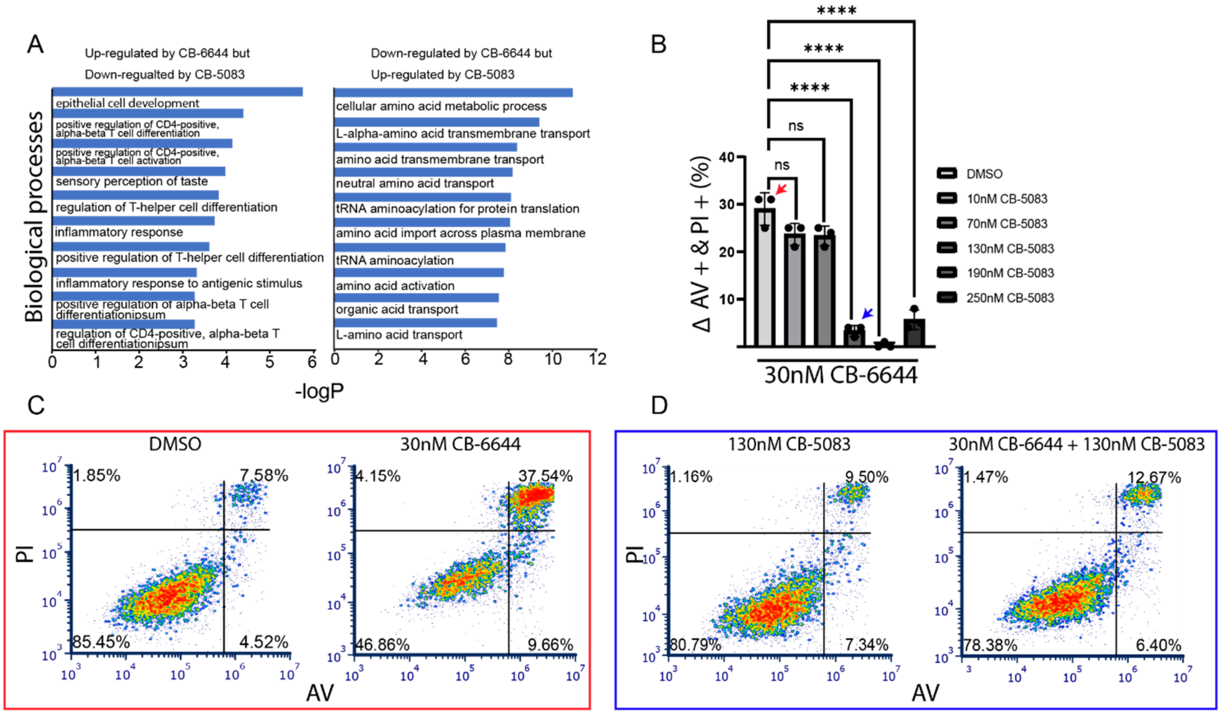

Figure S5: Experimental validation of predicted synergy of CB-6644 and CB-5083 in RPMI 8226 cells. (A) Gene ontology enrichment analysis on biological processes for leading genes identified from the GSEA analysis for those upregulated by CB-6644 but downregulated by CB-5083 (left panel) or for those downregulated by CB-6644 but upregulated by CB-5083 (right panel). (**B**) % of PI and AV double-positive cells from treatment with 30 nM CB-6644 combined with varying concentrations of CB-5083 in RPMI 8226 cells for 48 hours, in relative to CB-5083 alone or DMSO alone. *: $P < .05$. **** $P < .0001$ (t-test). ns: not significant. n = 3 for each condition. (**C**) Representative flow panel for the % of PI and AV double-positive cells, indicated as red arrowhead in panel **B**, from CB-6644 treatment (30nm) relative to DMSO alone (37.54%-7.58%). (**D**) Representative flow panel for the % of PI and AV double-positive cells, indicated as blue arrowhead in panel **B**, from the combined treatment of CB-6644 (30nm) and CB-5083 (130nm) relative to CB-5083 alone (130nm) (12.67%-9.50%). Live cells: lower left quadrant; apoptotic cells: lower right quadrant; dead cells: upper right quadrant.
